# Prognostic value of capillary refill time in adult patients: a systematic review with meta-analysis

**DOI:** 10.1186/s13054-023-04751-9

**Published:** 2023-12-02

**Authors:** Matthias Jacquet-Lagrèze, Aymeric Pernollet, Eduardo Kattan, Hafid Ait-Oufella, Delphine Chesnel, Martin Ruste, Rémi Schweizer, Bernard Allaouchiche, Glenn Hernandez, Jean-Luc Fellahi

**Affiliations:** 1 Service d’anesthésie-Réanimation, Hôpital Cardiologique Louis Pradel, 59 Bd Pinel, 69500 Hospices Civils de LyonBron, France; 2 Faculté de Médecine Lyon Est, Université Claude Bernard Lyon 1, 8, Avenue Rockefeller, 69373 Lyon Cedex 08, France; 3 CarMeN Laboratoire, Inserm UMR 1060, Université Claude Bernard, Lyon 1, Lyon, France; 4 Departamento de Medicina Intensiva, Facultad de Medicina, Pontificia Universidad Católica de Chile, Santiago, Chile; 5 The Latin American Intensive Care Network (LIVEN), Santiago, Chile; 6 Hôpital Saint-Antoine, Service de Médecine Intensive-Réanimation, Sorbonne Université, Paris, France; 7 Service d’anesthésie-Réanimation, Hôpital Lyon Sud, Hospices Civils de Lyon, 165 Chem. du Grand Revoyet, 69495 Pierre-Bénite, France

**Keywords:** Capillary refill time, Septic shock, Acute circulatory failure, Microcirculation

## Abstract

**Purpose:**

Acute circulatory failure leads to tissue hypoperfusion. Capillary refill time (CRT) has been widely studied, but its predictive value remains debated. We conducted a meta-analysis to assess the ability of CRT to predict death or adverse events in a context at risk or confirmed acute circulatory failure in adults.

**Method:**

MEDLINE, EMBASE, and Google scholar databases were screened for relevant studies. The pooled area under the ROC curve (AUC ROC), sensitivity, specificity, threshold, and diagnostic odds ratio using a random-effects model were determined. The primary analysis was the ability of abnormal CRT to predict death in patients with acute circulatory failure. Secondary analysis included the ability of CRT to predict death or adverse events in patients at risk or with confirmed acute circulatory failure, the comparison with lactate, and the identification of explanatory factors associated with better accuracy.

**Results:**

A total of 60,656 patients in 23 studies were included. Concerning the primary analysis, the pooled AUC ROC of 13 studies was 0.66 (95%CI [0.59; 0.76]), and pooled sensitivity was 54% (95%CI [43; 64]). The pooled specificity was 72% (95%CI [55; 84]). The pooled diagnostic odds ratio was 3.4 (95%CI [1.4; 8.3]). Concerning the secondary analysis, the pooled AUC ROC of 23 studies was 0.69 (95%CI [0.65; 0.74]). The prognostic value of CRT compared to lactate was not significantly different. High-quality CRT was associated with a greater accuracy.

**Conclusion:**

CRT poorly predicted death and adverse events in patients at risk or established acute circulatory failure. Its accuracy is greater when high-quality CRT measurement is performed.

**Supplementary Information:**

The online version contains supplementary material available at 10.1186/s13054-023-04751-9.

## Introduction

Acute circulatory failure results in tissue hypoperfusion that leads to life-threatening organ dysfunction. The prognostic value of tissue hypoperfusion has generated substantial research interest. Notably, the evaluation of peripheral perfusion through capillary refill time (CRT) has gained considerable attention during the last decade. CRT measures the amount of time necessary for the skin to return to baseline color after the application of a firm pressure (Additional file 1). CRT can be measured easily at the bedside within a few seconds, and there are more rapid changes after resuscitation when compared to lactate clearance [1]; variations of CRT after a passive leg raising [2] or a fluid challenge [3] can be detected within a few seconds. Furthermore, CRT measurement is an easy-to-use, costless method that allows tissue perfusion assessment at admission as well as during ICU stay. Since its first description [4], CRT became popular in the 1980s when Champion et al. included CRT in the Trauma Score [5]. Since then, CRT has been found to be able to assess severity [6–10] or to guide treatments [11] in different settings. In addition, a recent randomized trial suggested that a resuscitation strategy targeting CRT normalization may reduce morbidity and mortality in septic shock patients when compared to a strategy based on lactate clearance [12, 13]. CRT was then recommended as a potential therapeutic target by international experts for critically ill patients [14]. However, the relationship between CRT and outcome are still unclear as studies have reported conflicting results [15–17]. The only published meta-analysis was conducted in pediatric patients [18], and it is of note that pediatric intensivists seem more convinced of the prognostic accuracy of CRT than those treating adult patients [17, 19].

We therefore conducted a systematic review of studies evaluating CRT as a prognostic factor in adult patients and performed a meta-analysis to assess the ability of CRT to predict death or adverse events in a context of acute circulatory failure or in a patients at risk of acute circulatory failure.

## Methods

We conducted the study according to the Cochrane Handbook for Systematic Reviews of Diagnostic Test Accuracy [20] and existing guidelines for reviews of diagnostic accuracy studies [21]. The study was reported in accordance with the preferred reporting items for systematic reviews and meta-analyses (PRISMA 2020) statement [22] (Additional file 3: Table S1). This systematic review was prospectively registered on PROSPERO (CRD42022297158, submitted 02/27/2022) prior to initiating data extraction.

### Eligibility criteria

Clinical trials eligible for this meta-analysis were those that studied the prognostic value of CRT in a context of established acute circulatory failure or in a patient at risk of acute circulatory failure. We defined acute circulatory failure as the need for vasopressors or inotropes in combination with signs of hypoperfusion. If the inclusion criteria of the studied population aligned with this definition, the study was included and categorized as a population of patients with acute circulatory failure. We defined patients at risk of acute circulatory failure as those for whom the CRT was used as a triage method without restriction to patients in acute circulatory failure (e.g., first evaluation at the emergency department, rapid response team first evaluation, patients with trauma, etc.). The primary analysis outcome was death with no specific time frame after CRT measurement. Adverse events were defined as any unfavorable event explicitly labeled as such in the analyzed reports. This included outcomes such as admission to ICU, extended length of stay, and severe complications according to the Clavien-Dindo scale. Additionally, we accepted composite outcomes that included death as part of the definition of an adverse event. We excluded studies that concerned animals, studies not published in English language, letters, and reviews, studies that did not study the relationship between CRT and prognosis, studies in which CRT was performed with a device, and studies assessing localized perfusion such as free flap, or ischemic limb.

### Search strategy

Eligible studies were identified by searching the MEDLINE, EMBASE, and Google Scholar databases from inception to February 2022 with no language restriction and using the following keywords: “Capillary refill time” or “Capillary refill.” We also screened articles in the reference section of review articles and conducted a snowballing procedure to examine the references in those review retrieved through the systematic search. No language restriction was applied to the searches.

### Study selection

Two authors (MJL and AP) independently reviewed and screened the title and abstract of potentially relevant studies and determined final eligibility through examination of full texts. Disagreements that could not be resolved among the two authors through discussion were addressed by a third author (JLF). We included studies that provided information about CRT and the outcome in adults, irrespective of clinical situation. The studies had to provide the number of patients with normal and abnormal CRT and the number of patients with positive or negative outcomes (or the sensitivity, specificity, and prevalence) in each situation to calculate the number of true positives, true negatives, false positives, and false negatives. If such data were unavailable but were reported to have been collected by the authors, we emailed the corresponding author to obtain the data. A second email including co-authors was sent one month later, and in case of no reply after a month the study was excluded.

### Data extraction

We used a standardized form to extract (independently collected by MJL and AP) the following variables from the selected studies: the year of publication, name of journal, the methods used to perform the CRT (site of measurement on the skin, duration of compression, mode of compression, use of a stopwatch), results, the nature of the patient’s state of shock if the patient was in shock, but also the sample size, the number of true positives, true negatives, false positives and false negatives, as well as the area under the receiver operating characteristic curve (AUC ROC). When several CRTs were performed in the first days of resuscitation, we retained the one on the first day and, if the information was given, we selected the one performed after initial resuscitation. High-quality CRT measurement was defined as those corresponding to the mean of 2 or more CRT values made using a standardized compression and a stopwatch.

### Quality assessment

Two authors (MJL and AP), using the QUADAS-2 tool for assessing risk of bias in diagnostic accuracy studies, independently determined the quality of the included studies through examination of the full text. QUADAS-2 tool [23] encompasses four domains: patient selection, index test, reference standard, and flow and timing. We used the signaling questions to judge risk of bias and applicability concern. We constructed the flow diagram for the primary study, and judged bias and applicability. The risk of bias and applicability was assessed as high, low, or unclear.

### Statistical analysis

We estimated the pooled AUC ROC, sensitivity, and specificity for CRT as a predictor of death or adverse events in patients with acute circulatory failure and at risk of acute circulatory failure. As we anticipated a great between-study heterogeneity, a random-effects model was used to pool effect sizes. The Mantel–Haenszel estimator was used to calculate Q and τ^2^. We used Knapp–Hartung adjustments [24] to calculate the confidence interval (CI) around the pooled effect. The effect size was the diagnostic odds ratio (DOR). We did not use continuity correction except to calculate individual study results in which we used a continuity correction of 0.5 in studies with zero cell frequencies. A forest plot was built to summarize the effect size of each study and pooled results.

The primary analysis was the ability of abnormal CRT to predict death in patients with acute circulatory failure. Secondary analyses included the ability of CRT to predict death or adverse events in patients at risk or with confirmed acute circulatory failure. Secondary analysis included the accuracy (AUC ROC, sensitivity, specificity, DOR) of abnormal CRT to predict death or adverse event, or to predict acute kidney injury in patients with, or at risk of, acute circulatory failure. We also estimated the accuracy of lactate as a predictor of death or adverse events in patients with acute circulatory failure and at risk of acute circulatory failure when data were available, and compared its accuracy to that of CRT. We also sought to identify explanatory factors associated with better accuracy.

We used the Spearman correlation coefficient between sensitivity and false positive rate to detect a threshold effect. We conducted several sensitivity analyses in predefined subgroups of patients, analyzed subgroup differences using the Q test, and *P* values of the tests were provided. We compared studies conducted in an ICU setting to those in a non-ICU setting; studies with patients in septic shock to those without patients in septic shock; studies in which patients were in acute circulatory failure to those in which patients were not in acute circulatory failure; studies in which the location of CRT was a finger to those in which this was performed at another location; studies in which CRT was performed using a method to apply pressure on the skin in a reproductive manner to those in which this was not the case; and studies describing the use of a stopwatch to measure CRT to those which did not. We also added four subgroup post hoc analyses: We compared studies with high-quality CRT measurement to those with low-quality CRT measurement; studies predicting death to those predicting adverse events; studies with a low risk of bias to those with a high risk of bias; and studies with a CRT threshold at 3 s to those using other thresholds. To evaluate the risk of bias, we used the quality assessment of diagnostic accuracy studies QUADAS-2 scale [23]. We built a scoring system, where, for each of the four domains, zero points were given for low risk, two points for high risk, and one point for unclear risk for each item of the QUADAS evaluation, and we then summed the sub-scores to calculate the QUADAS score; studies at low risk of bias were those with a score below or equal to the median of all the scores, and studies at high risk of bias studies were those with a scores strictly greater than the median of all the scores.

In a sensitivity analysis, we also investigated the causes of heterogeneity using outlier detection. We defined outliers as studies that showed an effect size that was out of the 95%CI of the effect size of the complete analysis. After excluding outlier studies, we calculated the pooled effect size on the remaining studies. Lastly, we also plotted the overall effect and I^2^ heterogeneity of all meta-analyses that were conducted using the leave-one-out method [25]. We performed a meta-regression based on a mixed effect model, including the same criteria as for the subgroup analyses if the *P* value was less than 0.5 to respect the concept of parsimony. We performed a prediction test to assess the robustness of the effect size. Results were expressed as mean (95%CI) or as mean ± standard deviation (SD). We used R version 4.0.4 (R Core Team 2017, Vienna, Austria) to perform statistical analyses. The meta [26] and meta4diag [27] packages were used. All tests were two-sided, and a p value less than 0.05 was considered significant.

## Results

### Characteristics of included studies

**Fig. 1:**
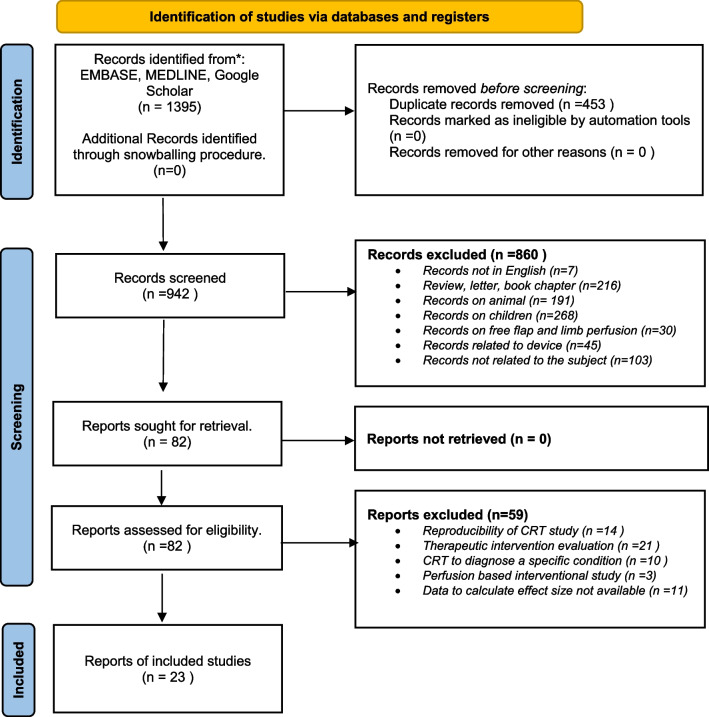
Flowchart of the meta-analysis selection process. *CRT* Capillary refill time

A total of 23 studies were included (Fig. 1), corresponding to 60,656 patients. These studies were published between 1994 and 2022; most of them (12/23) between 2019 and 2022. Investigations were performed in the emergency department (*n* = 8, 35%), in the ICU (*n* = 10, 43%), the operating room (*n* = 1, 4%), and the prehospital setting (*n* = 4, 17%). In 13 studies (57%), only patients with acute circulatory failure were included, and in 11 studies (48%) only those with septic shock were included. The characteristics of included studies are presented in Table 1. The mean ± SD abnormal CRT threshold value was 3.3 ± 0.8 s. The site of CRT measurement was the fingertip in 18 studies (78%), the chest in 2 studies (9%), and the knee in 3 studies (13%). A stopwatch was used in 12 studies (52%). High-quality CRT measurement was performed in 5 studies (22%). In 7 studies (30%), CRT was assessed before initial resuscitation. The mean ± SD frequency of the studied outcome (death or adverse event) was 26 ± 14%; that of death was 23 ± 14%. A summary of the sensitivity and specificity of CRT in individual studies is provided in Additional file 2: Figure S1.

**Table 1:** Characteristics of included studies

	Outcome	Type of adverse event	Time of outcome assessment	Setting	Circulatory failure	Septic shock	Abnormal CRT threshold
Lechleuthner et al*.* [44]	Adv. events	Uncontrolled bleeding	Hosp. stay	PH	NO	NO	2
Holcomb et al*.* [37]	Adv. events	Lifesaving intervention requirement	Hosp. stay	PH	NO	NO	2
Pealing et al*. *[45]	Adv. events	Death due to bleeding	Hosp. stay	ED	NO	NO	3
Ait-Oufella et al*.* [6]	Vital status		D14	ICU	YES	YES	4.9
Mrgan et al*.* [46]	Vital status		D7	ED	NO	NO	3
Van Genderen et al*.* [8]	Adv. events	According to the Clavien-Dindo classification	D10	OR	NO	NO	4.5
Hernandez et al*.* [28]	Vital status		Hosp. stay	ICU	YES	YES	4
Coslovsky et al*.* [29]	Vital status		Hosp. stay	ED	NO	NO	3
Bourcier et al*.* [47]	Vital status		Hosp. stay	ICU	YES	YES	3
Alegria et al*.* [48]	Vital status		Hosp. stay	ICU	YES	YES	3
Lara et al*.* [49]	Vital status		Hosp. stay	ED	YES	YES	3
Serano et al*.* [50]	Vital status		D30	ED	YES	NO	4.5
Jacquet-Lagreze et al*.* [17]	Vital status		D90	ICU	YES	NO	3.9
Darioli et al*.* [30]	Vital status		D2	PH	NO	NO	2
Jouffroy et al*.* [16]	Vital status		D38	PH	YES	YES	4
Mongkolpun et al*.* [51]	Vital status		D4	ED	YES	YES	4
Bige et al*.* [52]	Adv. events	Intra-hemodialytic instability defined as a blood pressure drop requiring therapeutic intervention	D0	ICU	NO	NO	3
Sebat et al*.* [53]	Vital status		Hosp. stay	ED	NO	NO	3
Amson et al*.* [54]	Vital status		D28	ICU	YES	YES	3
Magnin et al*.* [55]	Vital status		D14	ICU	YES	YES	3
Morocho et al*.* [31]	Vital status		D28	ICU	YES	YES	3.5
Rossello et al*.* [38]	Vital status		D30	ED	NO	NO	3
Lavillegrand et al*.* [56]	Vital status		ICU stay	ICU	YES	YES	3

	Location	Resuscitation status	Assessment timing	Compression technique	Duration of compression	Stopwatch	Number of measurements	Quality of CRT measurement	Sample size	Mortality rate,%
Lechleuthner et al*.* [44]	finger	Unclear	D0	NA	NA	NO	NA	Low	353	22%
Holcomb et al*.* [37]	finger	Unclear	D0	NA	NA	NO	NA	Low	216	6%
Pealing et al*. *[45]	chest	After	D0	NA	NA	NO	NA	Low	20,127	5%
Ait-Oufella et al*.* [6]	knee	After	D1	Blanch. nail	15	YES	4	High	59	37%
Mrgan et al*.* [46]	finger	before	D0	Firm press	5	YES	1	Low	1935	10%
Van Genderen et al*.* [8]	finger	After	D0	Firm press		YES	2	High	137	36%
Hernandez et al*.* [28]	finger	before	D0	Firm press	15	YES	NA	Low	104	31%
Coslovsky et al*.* [29]	finger	Unclear	D0	NA	NA	NO	NA	Low	8606	5%
Bourcier et al*.* [47]	finger	After	D0	NA	NA	NO	NA	Low	40	21%
Alegria et al*.* [48]	finger	After	D0	NA	NA	NO	NA	Low	90	10%
Lara et al*.* [49]	finger	After	D0	Firm press	10	YES	1	Low	100	14%
Serano et al*.* [50]	finger	Unclear	D0	NA	NA	NO	NA	Low	212	27%
Jacquet-Lagreze et al*.* [17]	chest	After	D0	Piston	7	YES	4	High	34	29%
Darioli et al*.* [30]	finger	before	D0	NA	NA	NO	NA	Low	11,639	5%
Jouffroy et al*.* [16]	finger	before	D0	NA	NA	YES	NA	Low	63	36%
Mongkolpun et al*.* [51]	finger	After	H6	Firm press	15	YES	1	Low	70	41%
Bige et al*.* [52]	finger	before	H0	Blanch. nail	15	YES	4	High	211	NA
Sebat et al*.* [53]	finger	before	H0	moderate press	5	NO	NA	Low	6480	36%
Amson et al*.* [54]	knee	After	D0	Firm press	15	YES	1	Low	64	34%
Magnin et al*.* [55]	knee	After	H24	Firm press	15	YES	1	Low	57	34%
Morocho et al*.* [31]	finger	before	H6	Firm press	10	YES	2	High	175	40%
Rossello et al*.* [38]	finger	Unclear	NA	NA	NA	NO	NA	Low	10,979	10%
Lavillegrand et al*.* [56]	finger	After	NA	NA	NA	NO	2	Low	30	33%

CI confidence interval, CRT: Capillary refill time, D: Day, ED: Emergency department, H: hour, Hosp. stay: Hospital stay, ICU: intensive care unit, NA: not available data, OR: Operating room, PH: prehospital

### Risk of bias and applicability concerns

**Fig. 2:**
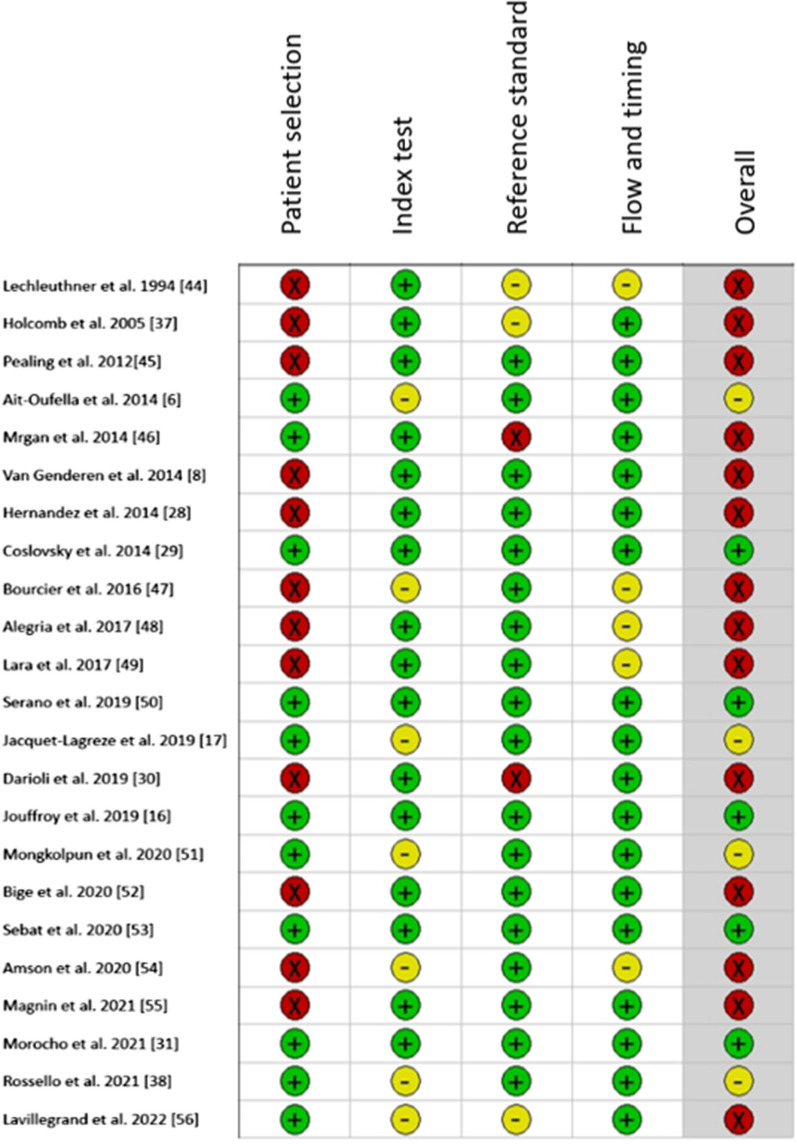
Light plot QUADAS evaluation of risk of bias of each study

**Fig. 3:**
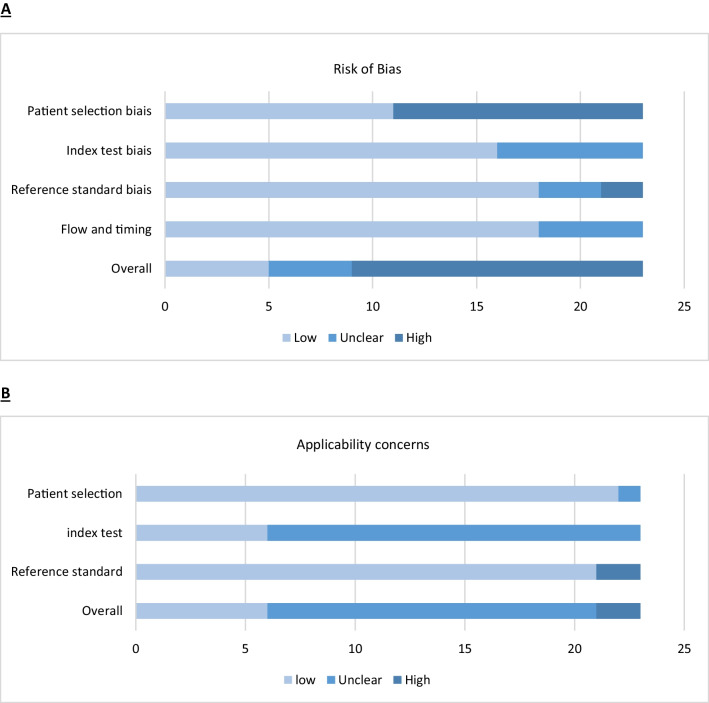
QUADAS assessment of risk of bias and applicability concern. Proportions of studies with low, unclear, and high risk of bias (**A**) or applicability concern (**B**) according to each item of the QUADAS evaluation

The overall risk of bias was high in 14/23 studies. (Individual study evaluations of the risk of bias are presented in Fig. 2, and pooled results in Fig. 3.)

### Primary analysis

Thirteen studies selected patients in acute circulatory failure and considered death as the outcome. In these studies, CRT was predictive of death; pooled AUC was 0.663 (95%CI [0.591; 0.756]). The pooled sensitivity was 54% (95%CI [43; 64]), and the pooled specificity was 72% (95%CI [55; 84]). The pooled DOR was 3.4 (95%CI [1.4; 8.3], *P* = 0.013; Table 2).

**Table 2:** Primary and secondary analyses

	Number of studies	Number of patient	AUC ROC	95%CI	OR	95%CI	Tau^2^	I^2^	P value
Primary analysis (Mortality in ACF patients)	13	1038	0.66	[0.59; 0.76]	3.4	[1.4; 8.3]	1.4	79%	0.013
Secondary analysis (Mortality or adverse event in patients at risk or confirmed ACF)	23	59,522	0.69	[0.65; 0.74]	4.3	[2.6; 7.3]	0.9	96%	< 0.001
Secondary analysis (Comparison of CRT and Lactate)									
*CRT*	9	7023	0.68	[0.60; 0.79]	3.2	[1.1; 9.1]	0.7	77%	0.687
Lactate	9	7023	0.54	[0.53; 0.55]	2.6	[1.3; 5.2]	0.8	81%	

*ACF* Acute circulatory failure, *AUC ROC* Area under the curve of the receiver operating characteristic CI Confidence interval, *OR* Odds ratio. *P* value stands for the *P* value the effect size with the random effect model for the two first analysis and the comparison of the effect size between lactate and CRT in the last analysis

### Secondary analysis

**Fig. 4:**
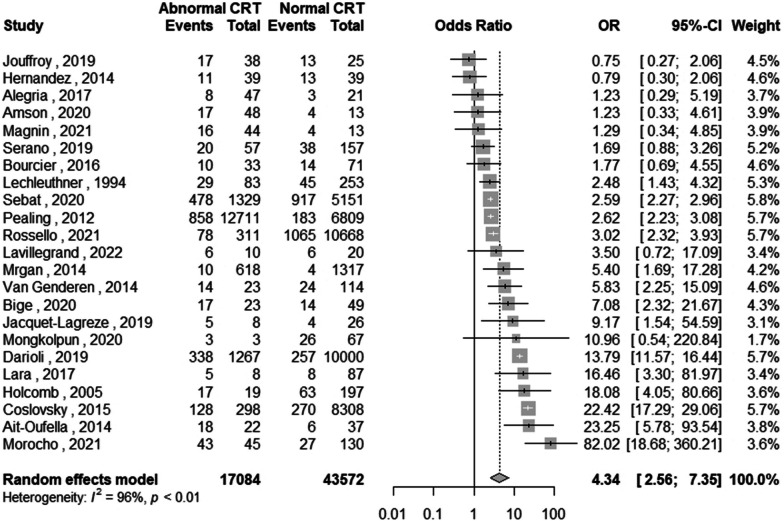
Diagnostic odds ratio of individual study and pooled odds ratio using a random effect model

In patients with acute circulatory failure or at risk of acute circulatory failure, CRT was also predictive of death or adverse events; the AUC was 0.69 (95%CI [0.65; 0.74]). The pooled sensitivity was 48% (95%CI [36; 61]), and the pooled specificity was 81% (95%CI [67; 90]). The pooled DOR was 4.3 (95%CI [2.6; 7.3], *P* < 0.001; Table 2, Fig. 4 and Fig S1.)

In patients with acute circulatory failure and at risk of acute circulatory failure (*n* = 11 studies), the arterial lactate level was not an accurate predictor of death; the AUC was 0.539 (95%CI [0.529; 0.549]). The pooled sensitivity was 46% (95%CI [18; 77]), and the pooled specificity was 76% (95%CI [52; 90]). The pooled DOR of an abnormal lactate to predict death or adverse events was 2.6 (95%CI [1.3; 5.2]; Table 2). After retrieving the information in reports and emailing the authors, we were able to compare CRT and lactate in 9 studies. Among the predictive ability of lactate and CRT in the 9 studies where both lactate and CRT were available, there was no significant difference between CRT and lactate to predict death (*P* = 0.687; Table 2).

The planned secondary analysis on acute kidney injury was not performed as only one studied reported this outcome but was among the 11 studies excluded due to the lack of data to assess the effect size.

### Heterogeneity and the causes of heterogeneity

#### I^2^ and prediction interval

The between-study heterogeneity I^2^ value was 96% (95%CI [95; 97]; details for heterogeneity in primary, secondary analyses, subgroup analyses, and sensitivity analyses are presented in Tables 2, 3, and 4, as well as in Additional file 2: Figure S2. The prediction interval ranged from OR = 0.5 to 34.6; as this includes 1, it indicates that due to varying effects, we cannot rule out that future studies may not confirm the diagnostic ability of CRT (Additional file 2: Figure S3). The correlation between sensitivities and false positive rates suggested a threshold effect (Spearman’s correlation coefficient: 0.68, 95%CI [0.37; 0.85]).

**Table 3:** Influence case removed analysis

	Number of studies	Number of patients	AUC	95%CI	OR	95%CI	p	95%PI	I^2^
Main Analysis	23	40,365	0.69	[0.65; 0.74]	4.3	[2.6; 7.3]	< 0.0001	[0.5; 34.6]	96%
Infl. Cases Removed^1^	18	20,195	0.67	[0.57; 0.82]	3.1	[2.2; 4.2]	< 0.0001	[1.7; 5.5]	55%

Removed as outliers: Hernandez, 2014 [28]; Coslovsky, 2015 [29]; Darioli, 2019 [30]; Jouffroy, 2019 [16]; Morocho, 2021[31]

*AUC* Area under the curve, *CI* Confidence interval, *OR* Odds ratio, *PI* Prediction interval

**Table 4:** Subgroups analyses

	Subgroups	Number of studies	Number of Patients	AUC ROC	95%CI	OR	95%CI	Tau^2^	I^2^ (%)	*P* value
Quality of CRT	*High*	5	451	0.84	[0.81; 0.87]	13.9	[3.6; 53.3]	0.76	64	0.009
	*Low*	18	60,109	0.72	[0.71; 0.72]	3.3	[1.9; 5.6]	0.94	97	
Stopwatch used	*YES*	12	2816	0.77	[0.75; 0.80]	5	[1.9; 12.7]	1.72	80	0.686
	*NO*	11	57,744	0.72	[0.72; 0.72]	4	[2.0; 7.9]	0.92	98	
Location of CRT	*Finger*	18	40,829	0.72	[0.72; 0.72]	4.6	[2.5; 8.5]	1.16	96	0.663
	*Other*	5	19,731	0.71	[0.66; 0.75]	3.5	[0.8; 15.8]	0.66	71	
Reproducible compression technic used	*YES*	3	165	0.83	[0.76; 0.90]	10.8	[2.2; 54.0]	0	0	0.025
	*NO*	20	60,395	0.72	[0.72; 0.72]	3.8	[2.2; 6.8]	0.95	96	
Number of averaged CRT	*2 or more*	6	481	0.83	[0.80; 0.86]	11.3	[3.5; 36.5]	0.73	62	0.019
	*one*	17	60,079	0.72	[0.71; 0.72]	3.2	[1.8; 5.8]	0.95	97	
Outcome	*Vital status*	18	40,305	0.72	[0.72; 0.72]	4.2	[2.2; 8.2]	1.23	96	0.922
	*Adverse events*	5	20,255	0.70	[0.66; 0.75]	4	[1.7; 9.7]	0.2	66	
Septic Shock	*YES*	11	792	0.77	[0.74; 0.80]	3.4	[1.2; 10.1]	1.94	82	0.423
	*NO*	12	59,768	0.72	[0.72; 0.72]	5.3	[3.0; 9.3]	0.9	98	
Setting	*ICU*	10	670	0.82	[0.79; 0.85]	3.9	[1.3; 11.3]	1.74	81	0.705
	*Non ICU*	13	59,890	0.72	[0.72; 0.72]	4.8	[2.6; 9.0]	0.93	97	
Risk of bias and applicability concern	*High*	11	40,340	0.78	[0.77; 0.78]	3.7	[1.6; 8.8]	1.47	97	0.81
	*Low*	12	20,220	0.62	[0.61; 0.62]	4.2	[2.1; 8.6]	0.28	77	
Resuscitation status	*After resuscitation*	11	20,135	0.70	[0.66; 0.75]	4.9	[2.0; 11.6]	1.20	98	0.526
	*Other*	12	40,425	0.72	[0.72; 0.72]	3.5	[1.8; 6.9]	0.35	58	
Threshold	*3 s*	12	47,933	0.67	[0.67 0.67]	3.6227	[2.0; 6.7]	0.70	96	0.389
	*Other value*	11	12,627	0.80	[0.79; 0.81]	5.7	[2.1; 15.6]	1.7	92	

*AUC ROC* Area under the curve of the receiver operating characteristic curve, *CI* Confidence interval, *CRT* Capillary refill time, *ICU* Intensive care unit, *OR* Odds ratio

### Sensitivity analyses

Subgroup analyses confirmed the significance of the effect size in all subgroups (Table 4). We then tested the effect of removing outliers from the analysis. The studies reported by Hernandez et al. [28], Coslovsky et al. [29], Darioli et al. [30], Jouffroy et al. [16], and Morocho et al. [31] (Fig. S2) showed an effect size that was out of the 95%CI of the effect size of the complete analysis. These studies were therefore considered as outliers and excluded. The analysis performed in the 18 remaining studies found a pooled AUC ROC of 0.67 (95%CI [0.57; 0.82]). The pooled sensitivity was 46% (95%CI [18; 77]), and the pooled specificity was 75% (95%CI [52; 90]). The pooled DOR was 3.1 (95%CI [2.2; 4.2], *P* < 0.0001), and the prediction interval OR = 1.7 to 5.5, τ^2^ = 0.0647 and *I*^*2*^ = 55% (95%CI [23; 73]) (Fig. S3). We also performed an influence analysis (Additional file 2: Figure S2) using the leave-one-out method, and no study was found to modify the meta-analysis. Finally, we performed a meta-regression; the variables with a *P* value less than 0.5 and hence included in the model were the following: septic shock as an inclusion criterion in the study, quality of CRT measurement, number of measurements contributing to the mean CRT value, and compression method. The model was not significant (*P* = 0.181), and the test for residual heterogeneity was significant (*P* < 0.0001). None of the covariates included in the meta-regression were found to be a significant source of heterogeneity.

## Discussion

This meta-analysis showed an overall low predictive value of CRT on mortality or adverse events in adults, both in established acute circulatory failure and in patients at risk of it. Furthermore, CRT was found to be a useful parameter for assessing the patient severity in various settings.

The pooled AUC ROC curve indicated that CRT was poorly accurate, but a significant effect size was found in all the studied situations and sensitivity analysis confirmed the predictive ability of CRT in these situations. This is of little surprise, as the link between mortality and hypoperfusion is not straightforward and many competing factors could influence mortality as an outcome [32], and is supported by the AUC ROC of lactate levels to predict death that was close to that of CRT. It is also of note that there was no significant difference between the ability of CRT and lactate to predict adverse events or death, which is consistent with the equivalence or superiority of CRT as a target for therapeutic intervention [12, 13]. In this context, and owing to stress-related hyperlactatemia, as well as the numerous pitfalls in the interpretation of lactate and lactate clearance, the clinical relevance of using lactate as a potential target in shock seems to be questionable [33]. Another point is that mortality was used as the outcome criterion (reference standard) of the primary analysis. This can be considered as methodological strength as this reduces the risk of bias, but studies aiming to explore the association between perfusion variables and organ dysfunction may be more relevant than mortality [34]. Herein, we planned to study renal function yet only one report was identified; although not included in the review it was found that prolonged CRT on the sternum in 1003 patients admitted to ICU was associated with acute kidney injury [35]. This suggests that further studies could be of interest, allowing a quantitative approach to be used; for example, assessing the correlation between CRT and serum creatinine could explore a dose–response relationship, providing further evidence between skin hypoperfusion and organ hypoperfusion [36].

A limitation of the evidence included in this review is that the included studies had very heterogeneous effect size, characteristics, and designs. However, both the subgroup analyses and the meta-regression argued against the influence of heterogeneity on the results. As the prediction interval of the odds ratio included one, the inclusion of future studies in this meta-analysis may not confirm the diagnostic ability of CRT. Removing outliers led to a decrease in heterogeneity without affecting the pooled effect size. Still, this heterogeneity in effect size can be explained by the heterogeneity of the setting and CRT measurement method used in each study. Some studies took place in ICU [6], others in ED [16], and others in prehospital settings [37]; in addition, some contexts were not widely studied such as heart failure [38] or postoperative settings [8]. The method applied to assess CRT differed markedly regarding stopwatch usage, duration and amount of compression, site of measurement, threshold; in addition, many did not report this in detail and it is likely that practice varied within these studies, reflecting that reported in real-life clinical practice [17]. This is of importance as a lack of standardization increases the risk of measurement bias [39–41]. Limitations of the review process include the exclusion of studies not reporting sufficient data to calculate the effect size and for which the contacted authors did not provide the lacking data; nevertheless, the number of patients included in these 11 studies represented 4519 patients (*data not shown*) that would have represented only 7% of the total number of patients if these had been included. The choice to exclude studies reported only by abstracts, studies not published in English language, as well as unpublished studies may have also increased the risk of reporting bias, but this risk bias was reduced by prospective PROSPERO registration with a pre-specified primary and secondary analysis. Other limitations of the review process include the absence of best CRT threshold calculation as an insufficient number of thresholds for each published study were given. Also, a threshold effect was detected, reflecting heterogeneity in thresholds; a ROC curve analysis was performed because these provide an overall summary of prognostic test’s accuracy, independent of this effect [42].

Implications of the review for practice are the following. First, as high-quality CRT increased by more than fourfold, the DOR to predict mortality further efforts to standardize the measurement technique in clinical practice is warranted. This may also be the key to explain the discrepancy on reproducibility on previous studies. Second, the meta-analysis supports a statistically significant link between abnormal CRT and a poor outcome. As CRT is recognized for its ability to reflect skin blood flow [43], and considering that isolated cutaneous hypoperfusion, as seen during mild cold exposure, generally does not result in systemic consequences such as death or adverse events, the notable association between the outcome and CRT suggests that prolonged CRT may signal compromised tissue perfusion. Consequently, CRT can be considered as a warning signal of tissue hypoperfusion in patient at risk or confirmed acute circulatory failure in clinical practice.

In conclusion, this meta-analysis showed that overall the CRT poorly predicted death or adverse events in patients at risk or established acute circulatory failure. As any single variable approach the prognostic value remains low but is comparable to lactate levels. Its accuracy is greater when high-quality CRT measurement is performed, and thus, efforts should be focused on standardizing the technique in clinical practice.

### Supplementary Information

**Additional file 1**. Supplementary file.

**Additional file 2**. Supplementary Figures.

**Additional file 3**. **Table S1. **PRISMA 2020 Checklist.

## Data Availability

The data that support the findings of this study are available from the corresponding author, [MJL], upon reasonable request.
