# Supplementary material for: Prognostic value of capillary refill time in adult patients: a systematic review with meta-analysis
Source: Crit Care. 2023 Dec 2;27:473. doi: 10.1186/s13054-023-04751-9 (PMC10693708; doi:10.1186/s13054-023-04751-9)
Supplement: Supplementary file 1 — Additional file 1. Supplementary file. [file 13054_2023_4751_MOESM1_ESM.docx]

**Supplementary file 1**

Recent years have witnessed a growing interest in CRT. Capillary refill time is best defined as the time taken to regain its color for the skin after being blanched by a firm pressure.

Typically, it can have the following aspect depicted in the following picture:


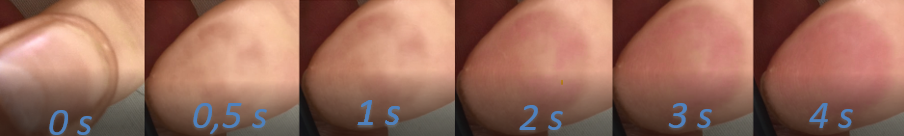


Some parameters must be considered to interpret CRT correctly:

Non modifiable factors

- Age[2]
- Sex [2]
- Underlying vascular disease[2]

Modifiable factors

- measurement site's elevation. Measuring CRT with the arm elevated above the bed may lead to underestimation.[3]
- temperature conditions
  - environmental [4]
  - patient temperature[5]
- lighting conditions [6]
- duration of compression. It is critical to maintain a compression duration of at least 7 seconds when assessing Capillary Refill Time (CRT). Prolonged compressions lasting up to 10 or 15 seconds have been commonly employed in most studies. [7,8]
- Ensure the application of standardized pressure on the skin by employing an appropriate method. Three distinct methods have been delineated for this purpose.
  - The microscope glass slide: “by applying firm pressure to the ventral surface of the right index finger distal phalanx with a glass microscope slide. The pressure will be increased until the skin is blank “[9]
  - The operator’s nail blanching crescent: “The pressure applied was just enough to remove the blood at the tip of the physician’s nail illustrated by appearance of a thin white distal crescent (blanching) under the nail.”[8]
  - The piston: “The piston characteristics were as follows: a 10-ml syringe filled in with 10 ml of air and closed with a plug We applied the piston on the skin; the 10 ml of air was compressed to fit a 7-ml volume, generating a pressure at the surface of the skin of 176 mmHg on a 2.5-cm2 surface.”[10]
- The use of a chronometer is warranted for precise measurement. [8–10]

**References**

1. Hernandez G, Boerma E, Dubin A, Pedreros C, Bruhn A, Koopmans M, et al. The relationship between microcirculatory flow abnormalities and systemic hemodynamic variables in septic shock patients. A multi-centre cross-sectional study [abstract]. Intensive Care Med. 2011;37:0341.

2. Schriger DL, Baraff L. Defining normal capillary refill: variation with age, sex, and temperature. Ann Emerg Med. 1988;17:932–5.

3. La Via L, Sanfilippo F, Continella C, Triolo T, Messina A, Robba C, et al. Agreement between Capillary Refill Time measured at Finger and Earlobe sites in different positions: a pilot prospective study on healthy volunteers. BMC Anesthesiol. 2023;23:30.

4. Gorelick MH, Shaw KN, Baker MD. Effect of ambient temperature on capillary refill in healthy children. Pediatrics. 1993;92:699–702.

5. Anderson B, Kelly A-M, Kerr D, Clooney M, Jolley D. Impact of patient and environmental factors on capillary refill time in adults. Am J Emerg Med. 2008;26:62–5.

6. Brown LH, Prasad NH, Whitley TW. Adverse lighting condition effects on the assessment of capillary refill. Am J Emerg Med. 1994;12:46–7.

7. Kawaguchi R, Nakada T-A, Oshima T, Shinozaki M, Nakaguchi T, Haneishi H, et al. Optimal pressing strength and time for capillary refilling time. Crit Care. 2019;23:4.

8. Ait-Oufella H, Bige N, Boelle PY, Pichereau C, Alves M, Bertinchamp R, et al. Capillary refill time exploration during septic shock. Intensive Care Med. 2014;40:958–64.

9. Hernández G, Ospina-Tascón GA, Damiani LP, Estenssoro E, Dubin A, Hurtado J, et al. Effect of a Resuscitation Strategy Targeting Peripheral Perfusion Status vs Serum Lactate Levels on 28-Day Mortality Among Patients With Septic Shock: The ANDROMEDA-SHOCK Randomized Clinical Trial. JAMA. 2019;321:654–64.

10. Jacquet-Lagrèze M, Bouhamri N, Portran P, Schweizer R, Baudin F, Lilot M, et al. Capillary refill time variation induced by passive leg raising predicts capillary refill time response to volume expansion. Crit Care. 2019;23:281.

- rage at least two measurements.[8–10]
