# Supplementary material for: Prognostic value of capillary refill time in adult patients: a systematic review with meta-analysis
Source: Crit Care. 2023 Dec 2;27:473. doi: 10.1186/s13054-023-04751-9 (PMC10693708; doi:10.1186/s13054-023-04751-9)
Supplement: Supplementary file 2 — Additional file 2. Supplementary Figures. [file 13054_2023_4751_MOESM2_ESM.docx]

**Figure S1: *Sensitivity and specificity of CRT in individual studies***


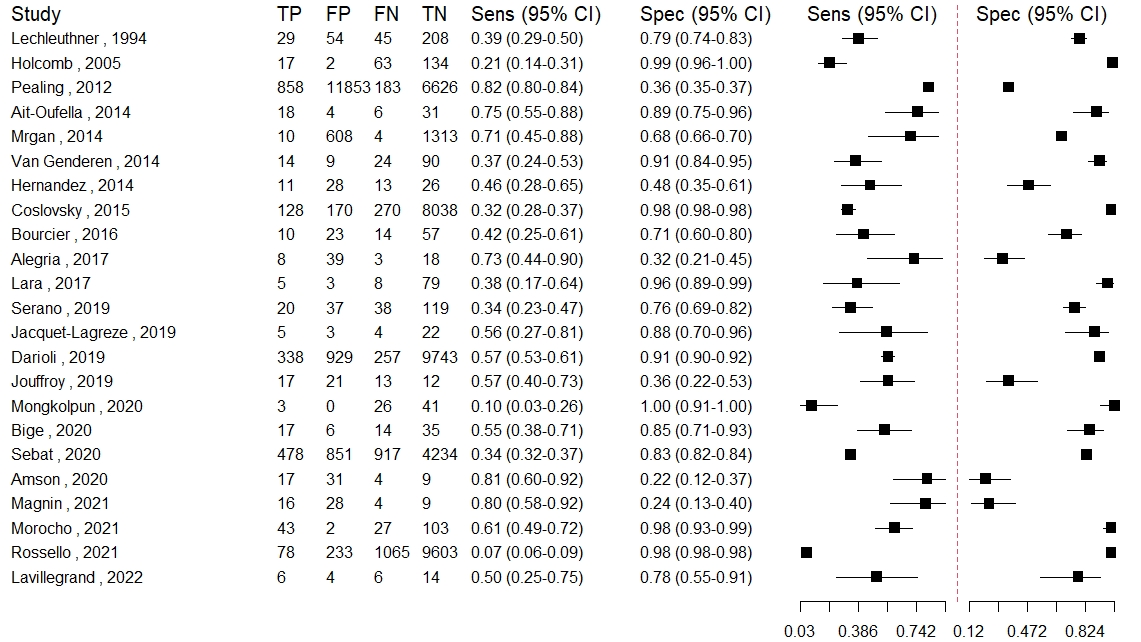


FN: False negative, FP: False positive, Sens: sensitivity , Spec: Specificity, TP: True positive, TN: True negative.

**Figure S2: *Influence analysis; leave-one-out meta-analysis.***

***A***


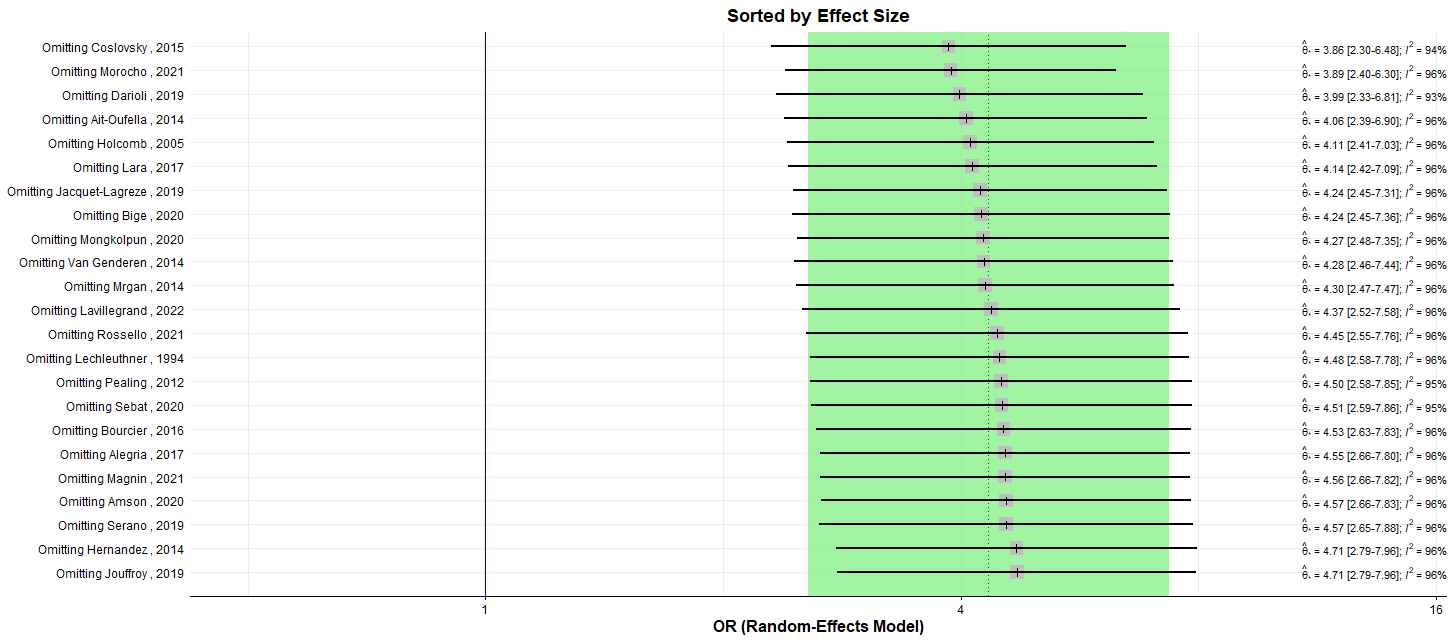


B


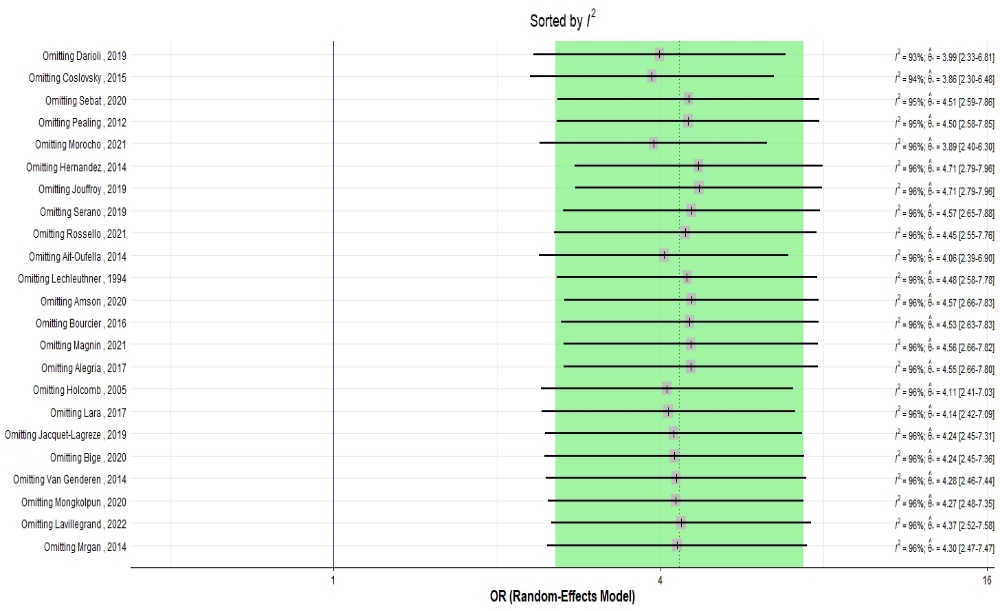


In Forest plot A and B are displayed the effect size ($\hat{\theta}$); grey squares with 95% confidence intervals (black horizontal line) and the heterogeneity (*I²*) with one study omitted each time. In plot (A), the meta-analyses are sorted by effect size, in plot B the meta-analyses are sorted by *I*². The dashed line and the shaded green area represent the initial effect size and the 95% confidence intervals of the pooled effect, before the leave-one-out method is applied.

**Figure S3: Drapery plot**


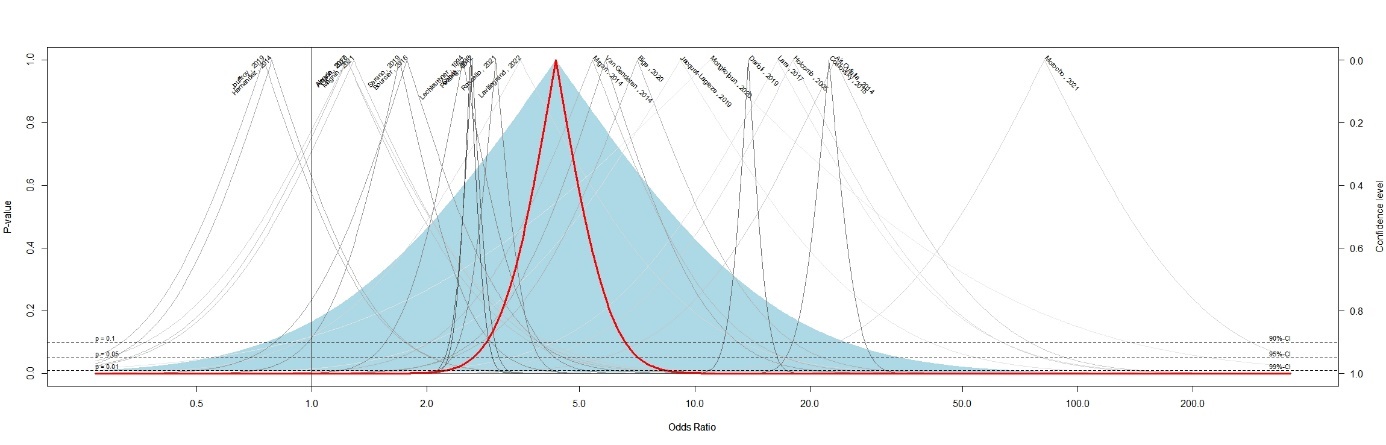


Drapery plot presents the P-value function as a curve for each individual studies. The thick red line represents the P-Value function of the pooled estimates according to the random-effects model which cross the OR 1 under the threshold P-Value 0.05. The blue shaded area represents the prediction interval, which cross the OR 1 above the P-Value 0.05.
